# Replanting

**Published:** 1881-06

**Authors:** J. H. Coyle

**Affiliations:** Thomasville, Ga.


### ARTICLE VI.

### *Replanting.*

BY J. H. COYLE, D. D. S., THOMASVILLE, GA.

It is not my purpose at this time to enter into an elaborate history of this operation, but only to give the results of my own experience, covering a period of over two years, and to give expression to my confidence in such practice. At that time it was altogether an experimental operation with me. It is true that I had seen the published expeience of others which had yielded a percentage of success far greater than that of the usual attempted treatment of such teeth *in situ.* On the other hand, I had read other articles which treated the operation with ridicule, and even went so far as to say that in transplanting, if we could get a tooth for the purpose, we could *shape and polish up an old tooth brush handle and insert that, and it would be a grand success.* Now, assertions like that were eminently calculated to throw doubt and mistrust on these operations and deter many dentists from trying the experiment themselves. The apparent hopelessness of some cases, however, those extreme cases of long standing abscess which so often weary out the operatnr and the patient, and oftentimes utterly refuse to yield to treatment, inspired me with the necessary courage to make experiments in this direction. The first patient was Dr.———, of this place. The four superior incisiors were badly decayed and filled, and the right lateral ulcerated and indicated a badly necrosed condition. The usual treatment was applied to this tooth for weeks without the slightest amendment, when I gave it up as a hopeless task. I then advised him to sacrifice the four incisors and replace with artificial teeth, but he would not do so. I then told him of the possibility of successful replanting, and he consented to try it at once. I extracted the tooth, cut off a small portion of the fang, cleansed the canal with carbolic acid, filled crown cavity and canal all with gutta percha, and filed a groove the entire length of the fang to allow any blood or serum in the alveolar to escape while pressing the tooth into place. When all was ready, I placed the tooth in a warm bath of warm water and carbolic acid, one part of the latter to thirty of the former, and after allowing it to remain a few minutes, took in my fingers and pressed it gradually and slowly into place, causing little or no pain. I then placed some warm gutta percha over the labial surface of tooth replaced, extending it over adjoining tooth on each side, pressing it well in between the teeth, and trimmed down with warm instrument and dismissed patient instructing him to call next day. When he presented himself the following day he said that he had some pain after leaving the office, and took a teaspoonful of paregoric, and had suffered no pain since. The second day after the operation, I removed the gutta percha and substituted a linen ligature, passing it alternately over and under the lateral and the adjoining tooth on either side, and directed him to touch the gums around the tooth three times daily with tincture myrrh. At the expiration of three weeks this tooth was as firm in its place as any tooth in his mouth, with abscess completely cured, and so remained. A year and a half afterwards, the two central becoming abscessed, he concluded to have the four extracted and replaced with plate, and when I extracted them I found this replanted lateral the hardest one of the four to remove—so firmly and uniformly had the periosteal union taken place; even the groove I filed on the fang was covered with periosteum.

Case 2. Mrs. R., of this place, first inferior left molar. Chronic abscess of long standing. Extracted it at once; cut off a small portion of each fang, treated as before, filed fangs with gutta percha, and crown with amalgam, and filed groove on each fang, extending up to the gum. When I came to press this tooth into the socket, I met with a good deal of resistance, on account of the convergence of the fangs toward each other, and so when it went home it did so in a hurry, not giving time for the passage of the serum in the alveolus up through the grooves in the fangs. The consequence was pain. I gave her forty drops of McMunn's Elixir Opium, with instructions to repeat it in four hours if necessary, which she did at bed time, and once the next day, and experienced very little discomfort. In ten days all soreness had disappeared, and this tooth is to-day just as firm and doing as good service as any tooth in her mouth, and gives not the slightest evidence of *abscess*.

Case 3. Col. M., of this place, presented with right superior lateral in a chronic state of abscess. Treated this tooth until the patient as well as myself became disgusted, when he consented to try the experiment of replanting. In the effort to extract it the crown was crushed. It was nothing but a shell, and this result was anticipated. I cleansed and filled the fang as usual, fitted a pivoted crown on it, and inserted it into its socket. In three weeks it was as firm as any other tooth, and the abscess had gone. A few days ago the pivot came out, and I repivoted it and found it just as firm and in as healthy condition as I could desire.

Case 4. Mrs. W., of this place, right superior lateral; abscess of long standing. Treated this tooth the usual way, curing the abscess, and inserted a pivot tooth thereon. I found after doing so that it projected a little too much, and in attempting to remove it for the purpose of correcting this defect, to my surprise, *the fang came out, too!* I took it into my laboratory, made the necessary change, replaced it, and to-day it is firm in its place, with no abscess, and doing good service.

And so I might go on and give other cases, but I fear it would lengthen this paper beyond the limits intended. My friend, Dr. McElvey, a practicing physician in an adjoining county, tells me that it is his custom on extracting teeth to replace them at once, if they are not too badly decayed. He simply breaks off the ends of the fangs with forceps, using them as a hammer, and without any other preparation presses them back into the sockets, and that he has twelve cases where they are firm, no abscess, and doing good service. I do not wish to be understood as advocating the practice in every case of abscessed teeth which present. Perhaps the majority of them will yield readily to proper treatment, but every practitioner of extended experience knows that there are cases which present no hope of a successful treatment dependent either upon special idiosyncrasy or circumstances, which make it impossible with the necessary treatment. Oftentimes one cause is residence a long distance from a dentist. And it is in cases of this kind that I recommend this practice. I believe that in forty-eight cases out of fifty, success will reward the effort, regard being had to the propriety of the operation in each case. I have not, up to this time, met with a single failure, and I therefore confidently assert that I have saved to my patients many valuable teeth by this practice, which would otherwise have been lost.—*Dent. Luminary.*

